# The nutritional quality and contents of heavy elements due to thermal processing and storage in canned *Thunnus tonggol* fish change compared to fresh fish

**DOI:** 10.1002/fsn3.3348

**Published:** 2023-03-28

**Authors:** Ali Aberoumand, Ferideh Baesi

**Affiliations:** ^1^ Department of Fisheries, Natural Resources Faculty Behbahan Khatam Alanbia University of Technology Behbahan Iran

**Keywords:** canned tuna, fresh tuna, heavy metals, pH, proximate composition, *Thunnus tonggol*

## Abstract

The purpose of this study was to evaluate the nutritional quality and concentration of heavy and toxic metals in the fresh and canned tuna *Thunnus tonggol* at different storage periods. The content of iron, zinc, copper, mercury, and also macronutrient compounds in the Iranian fresh and canned tuna fish and the effects of thermal processing and subsequent storage on metal contents were evaluated using atomic absorption spectroscopy. The results showed that the levels of iron, zinc, copper, and mercury after the 6th, 9th, and 11th months of storage were 26.52, 10.83, 6.22, and 0.04 mg/kg, respectively. The concentrations of iron, zinc, copper, and mercury in the fresh fish were 11.03, 7.11, 1.71, and 0.03 mg/kg, respectively. The results of the statistical analysis of the samples showed that canning process and sterilization by autoclave increased the contents of elements except mercury to a significant level (*p* < .05). The results showed that the amount of fat significantly increased in all samples after storage (*p* < .05), but the ash and protein content significantly decreased (*p* < .05). The moisture content significantly increased (*p* < .05) except for the 9th month of storage. The obtained results showed that the energy value after 6 months of storage was the highest (297.53 kcal/100 g). The results showed that the bioaccumulation of copper, iron, zinc, and mercury in the fresh and canned muscles was lower than the standard concentration recommended by the FAO and WHO. This type of fish was a high‐quality food source and it was safe after 11 months of storage and was suitable for human consumption. Therefore, the consumption of Iranian canned tuna can be safe for human health despite the possible contamination with heavy metals.

## Graphical abstract

The results of the statistical analysis of the samples showed that canning and sterilization by autoclave increased the contents of elements except mercury to a significant level (*p* < .05). The results showed that the amount of fat significantly increased in all samples after processing (*p* < .05), but the amount of ash and protein significantly decreased (*p* < .05). The moisture content significantly increased (*p* < .05) except for the 9 months of storage after canning. The obtained results showed that the energy value after 6 months of storage was the highest (297.53 kcal/100 g). The results showed that the bioaccumulation of copper, iron, zinc, and mercury in the fresh and canned muscles was lower than the standard concentration recommended by the FAO and WHO. This type of fish was a high‐quality food source and it was safe after 11 months of storage and was suitable for human consumption. Therefore, the consumption of canned Iranian tuna can be safe for human health despite the possible contamination with heavy metals.

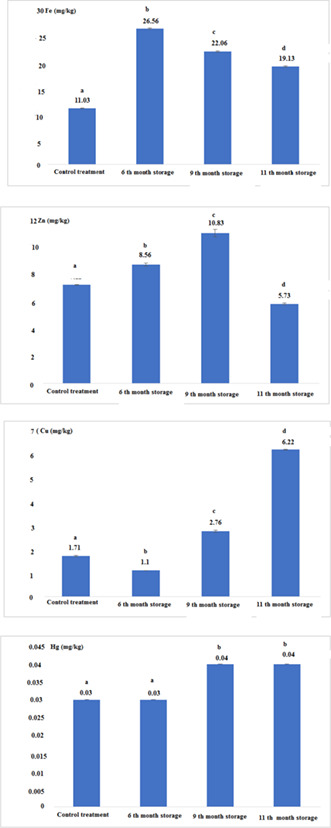

## 1 INTRODUCTION

Today, the distribution and consumption of canned products have been widespread because of easy usage of canned fish (Fisheries Research Institute of Iran, 2007). This product may be contaminated because of the high consumption of this product due to the use of metal cans for packaging (Bonyadian et al., 2012). There is a possibility of quality problems and pollution in this valuable food source due to the increasing pollution of aquatic ecosystems (Cheung & Chan, 1999).

Rahimi et al. (2014) investigated the determination of arsenic concentration in canned tuna in Isfahan and Shahrekord in Iran. In his study, the concentration of arsenic in 60 canned tuna was measured. Arsenic concentrations in canned tuna samples were lower than the EU standard. Ghazi Khansari & Afshar (1996) studied the concentration of heavy metals in tuna fish. Their results showed that the amount of accumulation of metals such as cadmium, arsenic, lead, mercury, and tin was lower than global standards. Lashkari Moghadam et al. (2010) conducted a study on the accumulation of heavy metals in canned tuna fillets and oil and found that the amount of nickel in tuna muscle was low but its amount was high in canned fish oil. The amount of cadmium in the product was more than the standard. Although the consumption of some nutritious fish is high in the world, due to the bioaccumulation of lead (Pb), cadmium (Cd), mercury (Hg), and other heavy metals that enter the fish muscles from the environment, it can have adverse effects on human health. These three metals are considered as one of the most important pollutants in the aquatic environment of fish, which is due to toxicity and accumulation in marine organisms and due to high durability in the environment (Castro‐González & Méndez‐Armenta, 2008). Although heavy metals are natural pollutants in the aquatic environment, their levels have increased due to industrial activities (Sarmiento et al., 2011). Mercury is one of the most abundant toxic metals and is a particularly important element because its mineral form is biologically converted in aquatic environments and even low concentrations of metals may threaten the health of aquatic organisms and humans (Olmedo et al., 2013).

One of the indicators for evaluating the nutritional value of fish is the determination of the percentage of proximate such as moisture, fat, protein, and ash. The proteins in fish tissue due to their essential amino acids have better nutritional value than the meat proteins (Alipour et al., 2011). The Qomi Behbahani and Javaheri Baboli (2014) showed that the percentage of protein and fat in the canned fish increased compared to fresh fish (control) and the percentage of moisture decreased significantly. Naseri et al. (2011) showed that the amount of fat, protein, moisture, and ash in fresh silver carp was significantly different from the canned fish. In this experiment, the percentage of moisture and fat decreased during the sterilization process and the percentage of protein and ash increased.

Fathi Achaloui and Abroumand (2015) showed that the amount of fat, protein, ash, and energy value in canned *Katsuwonus pelamis* changed significantly during the 3 months of storage. In *Euthynnus affinis* also, the protein percentage also changed significantly. In this experiment, there was no significant difference in pH between both canned fish species during storage. Honarvar (1999) stated the reason for the increase in the percentage of protein during the canning process was heat treatment in the preliminary stage and sterilization, which caused the separation of moisture from the fish tissue and ultimately increased the percentage of dry matter and protein content. Moini et al. (2008) showed the increase in the percentage of fat in canned fish compared to the control sample was the thermal process performed on the fish tissue, because during this process a high percentage of moisture in the fish was lost, which increased the percentage of dry matter and fat content. Another reason for the increase in the percentage of the fat in canned food was the addition of oil to the canned food during processing. Baygar and Gökoğlu (1999) investigated on fresh and canned Hoover fish for pH. Their results showed that the pH of fresh fish was 5.62 and canned fish was 5.83. The purpose of this study was to evaluate the nutritional value and the heavy metals levels in the canned tuna fish compared to the fresh tuna fish. While canned fish represents a popular and nutritious delicacy globally, frequent consumption may just conceivably infer a risk to health, some particular elements that can build up to toxic levels in body stores within the body. Contamination of toxic heavy metals in fish is a common problem worldwide. Trace elements in fish may come from various sources available in the aquatic environment, including within the water body itself, stored in the sediment and/or the terrestrial environment, the dietary habit of the fish, the preservation process and handling, etc. Toxic heavy metals are understood to enter the aquatic food chain via both the dietary (direct consumption of water and biota) and non‐dietary (uptake through absorbing epithelia in fish) routes; thus, aquatic organisms accumulate metal concentration several fold greater than that of the surrounding medium, and become an important media to transfer toxic metal from one trophic level to another.

Many studies have pointed to adverse effects on human health that may occur through the consumption of fish contaminated with trace metals, and some known diseases have been associated with trace metals. For instance, mercury has been implicated in neurological effects, while copper has been connected to anemia. Zinc is an essential element but at high concentrations, it leads to lung diseases, gastroenteritis, fever, vomiting, muscular coordination problem, and dehydration. In the process of canning of fish, the level of toxic elements will become more concentrated and the probabilistic health effect in consumption of those fish may increase many fold. The number of studies have been conducted on toxic heavy metals in different edible fish species. As canned fish occupies a special place in the diet and in some societies consumed frequently, there is a need to determine the level of heavy metals in canned fishes, and assess the concomitant risk associated with their dietary intake. With this in mind, the main aims of this study are to determine the concentrations of selected heavy metals such as copper (Cu), and zinc (Zn) in the more largely consumed species of canned fish available in Iran, and also to assess the associated risk to human health via their consumption.
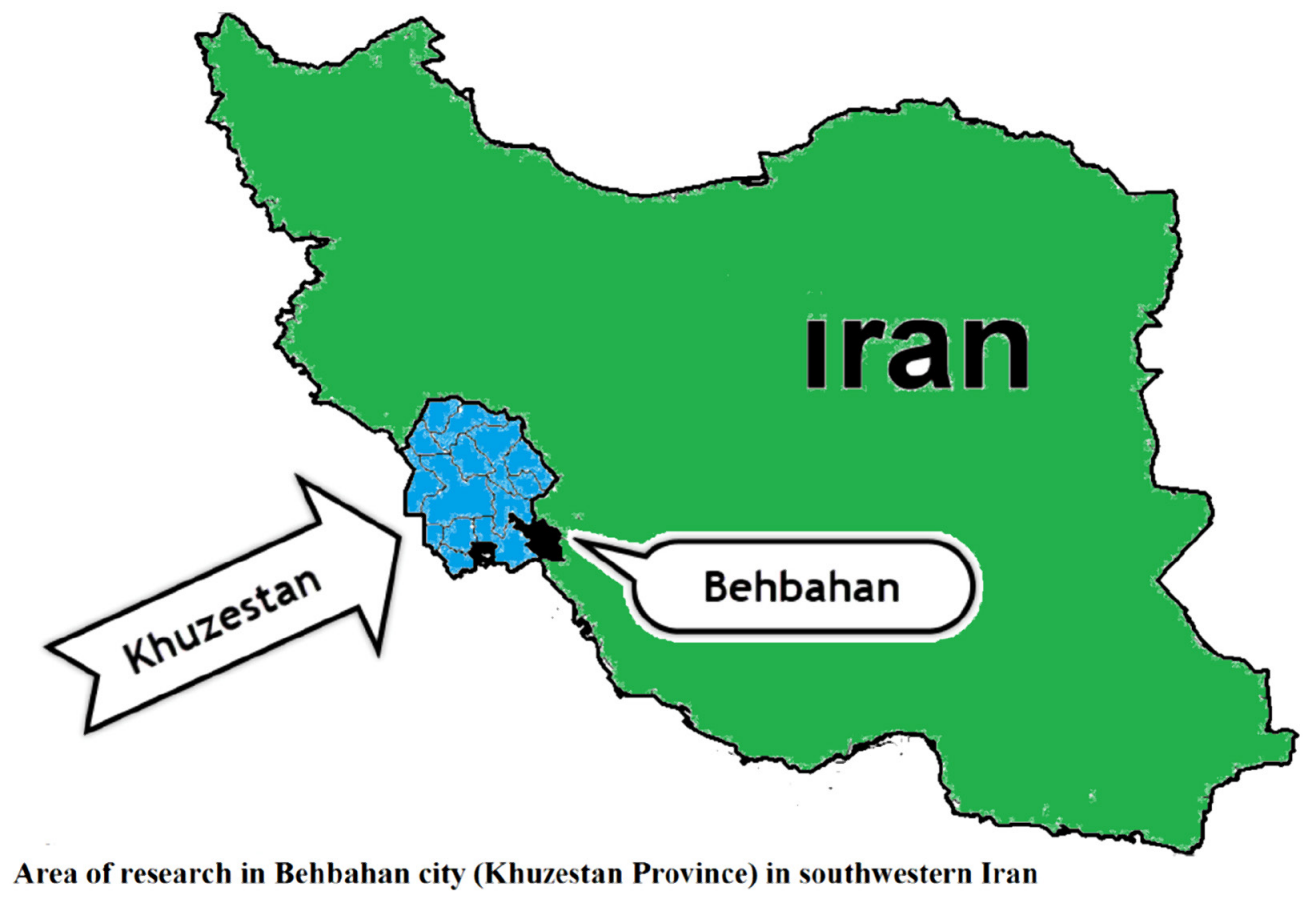
Study area map

## 2 MATERIALS AND METHODS

### 2.1 Preparation of samples

This present research has been done in 2021 in the fisheries laboratory of Khatam Alanbia University of Technology in Behbahan, Iran. To prepare the samples, 15 canned tuna and 5 kg of the fresh tuna fish (*Thunnus tonggol*) were purchased from Behbahan city market. The tuna were placed completely in clean plastic bags. After transferring the samples to the fisheries laboratory, all samples were washed thoroughly with distilled water to remove viscous substances and adsorbent particles from the fish's body surface. First, the head, tail, and internal organs of the fish were removed with a knife, and then the muscle fillets were separated and transferred to pre‐cleaned containers.

The fresh tuna fish was only used as the control and not stored. The canned tuna fish were stored separately at room temperature for 6, 9, and 11 months and then analyzed. No oil was added to canned tuna after 6 months of storage. The fresh and canned fish after 6, 9, and 11 months of storage were transferred to the laboratory in Behbahan city, Iran for analysis of proximate compositions and heavy metals. The proximate compounds (fat, protein, moisture, ash contents, and energy values), pH, and heavy metals such as copper, zinc, iron, and mercury were measured. All used materials in the present study had certified references.

### 2.2 Measurement of the moisture of the fish

To measure the percentage of moisture in the fresh tuna fillets and canned fish fillets, 10 g of the sample was weighed, and then, it was placed in a container. The prepared samples were placed in an oven at 103°C until reaching a constant weight. After leaving the oven, the container was placed in a desiccator for 30 min to cool and then weighed (AOAC, 2005). Moisture percentage was determined using the following equation:

$$\begin{array}{c} \text{Percentage of dried matter}=\\ \;\left(\text{Weight of dried sample}/\text{Initial weight of sample}\right)\times 100 \end{array}$$

$$\begin{array}{c} \text{Percentage of moisture}=\\ \;\text{Percentage of}\;\mathrm{wet}\;\text{matter}-\text{Percentage of dried matter} \end{array}$$

### 2.3 Measurement of protein percentage

The protein percentage of the samples was determined using the Kjeldahl method. One gram of each sample was placed in a digestion flask and then 150 mL of concentrated sulfuric acid was added to each flask along with the catalyst. The balloons in the desired device were placed to boil at a low temperature for 30 min until the foam comes out. After that, the temperature was increased to digest the sample, which took time about 4 h. After cooling the samples, distilled water was added to each balloon and placed in the titration section of the device. The samples were titrated with 1% normal sulfuric acid. The percentage of crude protein was obtained by determining the amount of total nitrogen using the formula 25.6 × % N (AOAC, 2005). The number obtained indicated the amount of crude protein. The percentage of nitrogen was also determined using the following equation:

$$\begin{array}{c} \text{Percentage of nitrogen}=\\ \;\text{Amount of acid consumption}\;0.1\;\text{normal}/\text{Sample weight}\times 100 \end{array}$$

### 2.4 Measurement of fat percentage

To measure the percentage of crude fat in the samples, 5 g of fresh and canned fish samples was wrapped in filter paper and placed in the extractor of the Soxhlet apparatus. Then, 100 mL of ether was placed into a balloon and connected to the refrigerant. Extraction was performed in a heater at a temperature of 60°C for 8 h. Distillation of the solution continued until the solution was free of solvent (AOAC, 2005). The prepared samples were then dried under the hood. The fat percentage of the samples was calculated using the following formula:

$$\begin{array}{c} \text{Percentage of}\;\mathrm{fat}=\text{Weight of the final sample}\\ \;-\text{Initial weight of the sample}/\text{Initial weight of the sample}\times 100 \end{array}$$

### 2.5 Measurement of ash percentage

An electric furnace was used to determine the percentage of ash in the samples. Ten grams of the samples already dried in the oven was placed in a container and then placed in an electric oven at 500°C for 12 h. The container was placed in a desiccator for 30 min to cool the samples (AOAC, 2005). The amount of gray sample remaining in the container was the amount of sample ash, which is obtained based on the following equation:

$$\text{Percentage of}\;\mathrm{ash}=\left(\text{weight of}\;\mathrm{ash}/\text{initial weight of sample}\right)\times 100$$

### 2.6 Measurement of energy values

The values of the fillet energy using the method of Schulze et al. (2005) were calculated. In this method, the total energy obtained from the protein and fat contents of the sample was determined based on the following relationship:

$$\begin{array}{c} \text{Energy level}\;\left(\mathrm{kJ}\;\mathrm{per}\;100\;g\;\text{of fillet}\right)=\\ \;\left(\text{Percentage of protein}\times 6.23+\text{Percentage of}\;\mathrm{fat}\times 8.39\right) \end{array}$$

### 2.7 Measurement of pH

Five grams of fresh and canned fish samples was mixed with 45 cc of distilled water. The homogenized sample was measured using a digital pH meter and its pH was determined (Masniyom et al., 2005).

### 2.8 Measurement of heavy metals

In order to chemically digest and measurement of concentration of heavy metals after transferring the samples to the laboratory, all samples were placed in an oven at 65°C for 150 min until they reached a constant weight. The dry method was used to digest the samples. Then, 0.5 g of the weighed sample was placed in a 250 mL flask, and 25 mL of concentrated sulfuric acid, 20 mL of 7 M nitric acid, and 1 mL of 2% sodium molybdate solution were added. For uniform boiling of the solution, several welding stones were placed in a balloon. After cooling, 20 mL of a mixture of concentrated nitric acid and concentrated perchloric acid in a ratio of 1:1 was added to the sample from the top of the refrigerant. The mixture is heated until the white vapors of the acid have completely disappeared. Slowly 10 mL of distilled water was added to the cooled mixture while the balloon is rotating. A completely clear solution is obtained by heating. After cooling, this solution was transferred to a 100 mL balloon and until it reached volume (Eboh et al., 2006; Kalay & Bevis, 2003). Heavy metals were measured by atomic absorption using Perkin Elmer 4100. To measure heavy metals, first 10 mL of the digested solution and 5 mL of 5% ammonium pyrrolidine carbamate solution were added and then the sample was shaken by a shaker for 20 min until the elements were complexed in metallic organic form in the solution. Then 2 mL of methyl isobutyl ketone was added to the solution and mixed for 30 min. After 10 min, the samples were centrifuged at 2500 rpm and the elements were transferred to the organic phase. After adjusting the furnace and EDL system (source of cathode ray production) of the device and optimizing the atomic absorption device, the calibration curve of these elements was plotted by Win Lab 32 software with the help of their standards and the modifiers of palladium and then the concentration of heavy metals in ready solutions measured (Olowu et al., 2010).

### 2.9 Statistical analysis of data

Two‐way ANOVA model with interaction was used to evaluate the data and in this study, data analysis was performed using SPSS software program (SPSS version 18.0). The significance level of 0.05 was applied to all statistical tests. Kolmogorov–Smirnov and Levine's tests were used for normality and homogeneity of variances. The mean of treatments was compared using *t*‐test and the presence or absence of significant differences was determined at the level of 5%. Excel 2003 software was used to draw the Figures.

## 3 RESULTS AND DISCUSSION

**FIGURE 1:**
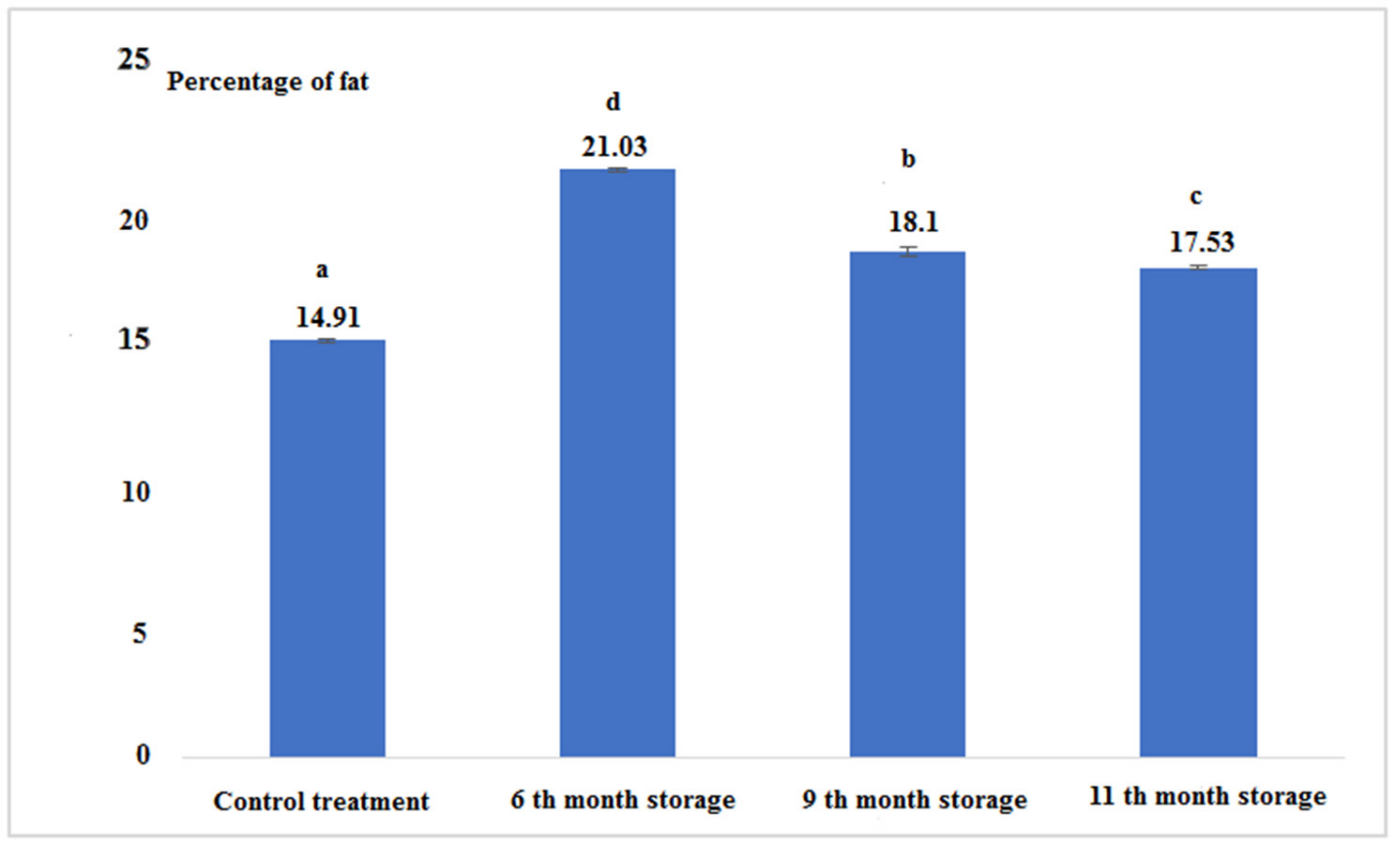
Percentage of the fat in fresh and canned tuna fillets.

According to Figure 1 and Table 1, it can be stated that the percentage of fat in canned fish after 6 months of storage was the highest comparing to the other experimental groups. The lowest was found for the fresh tuna. The results showed that the percentage of fat in all experimental treatments had a significant difference from each other (*p* < .05).

**TABLE 1:** Nutrient composition (%), energy level (Kcal/100 g), and pH of fresh and canned tuna fillet during different periods of storage.

Treatments	Fat	Protein	Moisture	Ash	Energy (kcal/100 g)	pH
Control (Fresh)	14.91 ± 0.04^a^	22.66 ± 0.57^a^	52.50 ± 0.26^a^	1.11 ± 0.00^c^	266.30 ± 3.33^a^	5 ± 0.00^a^
6th‐month storage	21.03 ± 0.05^d^	19.43 ± 0.37^b^	57.50 ± 0.17^b^	0.81 ± 0.02^b^	297.53 ± 2.68^b^	5 ± 0.00^a^
9th‐month storage	18.1 ± 0.17^b^	18.53 ± 0.23^c^	50.33 ± 0.57^c^	0.63 ± 0.01^a^	267.15 ± 2.61^a^	5.1 ± 0.00^b^
11th‐month storage	17.53 ± 0.05^c^	18.13 ± 0.05^c^	59.0 ± 0.08^d^	1.22 ± 0.02^d^	259.69 ± 0.67^c^	5 ± 0.00^a^

*Note*: The same letters in each column indicate the absence of significant differences (*p* < .05).

**FIGURE 2:**
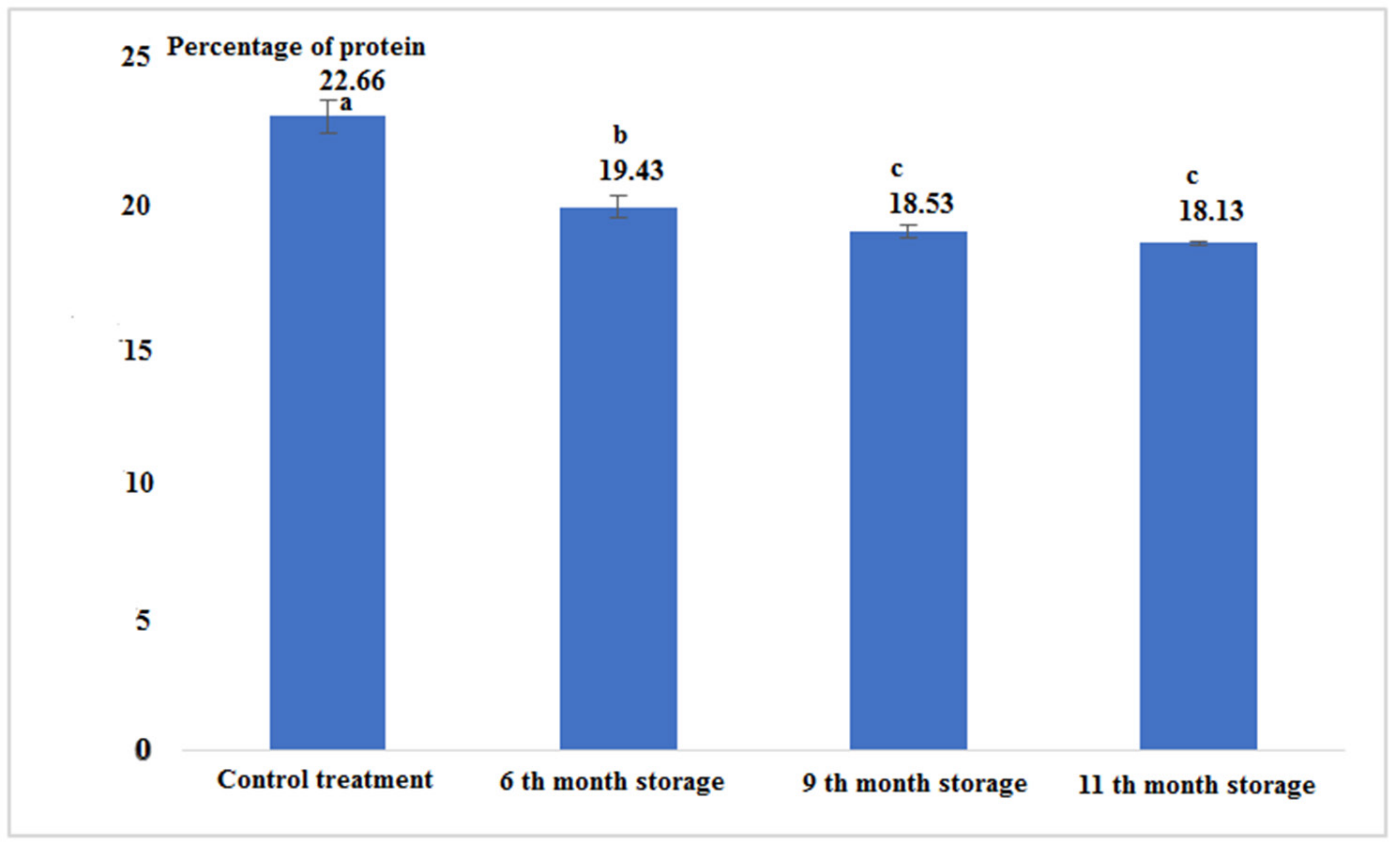
Percentage of protein in fresh and canned tuna fillets.

Fresh tuna fish contains a percentage of protein more than canned fish. The results showed that during the canning process and after it, the protein content of tuna fillets decreased significantly. The canned fish in the 9th and 11th month of storage had no significant difference, but in comparison with other treatments had a significant difference (*p* < .05; Figure 2 and Table 1).

**FIGURE 3:**
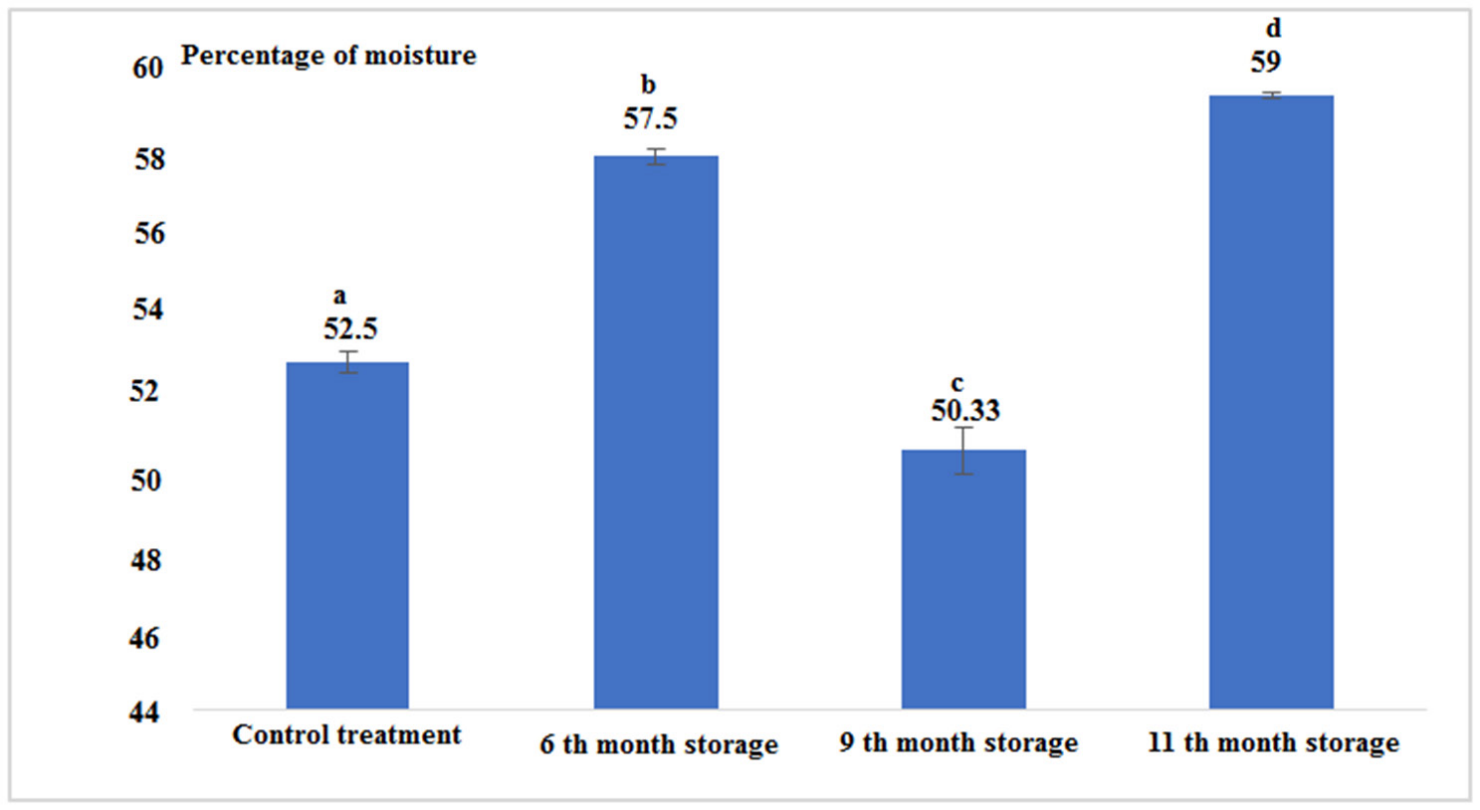
Percentage of moisture in fresh and canned tuna fillets.

According to Figure 3 and Table 1, it can be stated that the percentage of moisture in all experimental treatments had a significant difference from each other (*p* < .05). The highest percentage of moisture (59%) was observed for canned fish in the 11th month after production and the lowest percentage in the 9th month of storage was 50.33% (*p* < .05).

**FIGURE 4:**
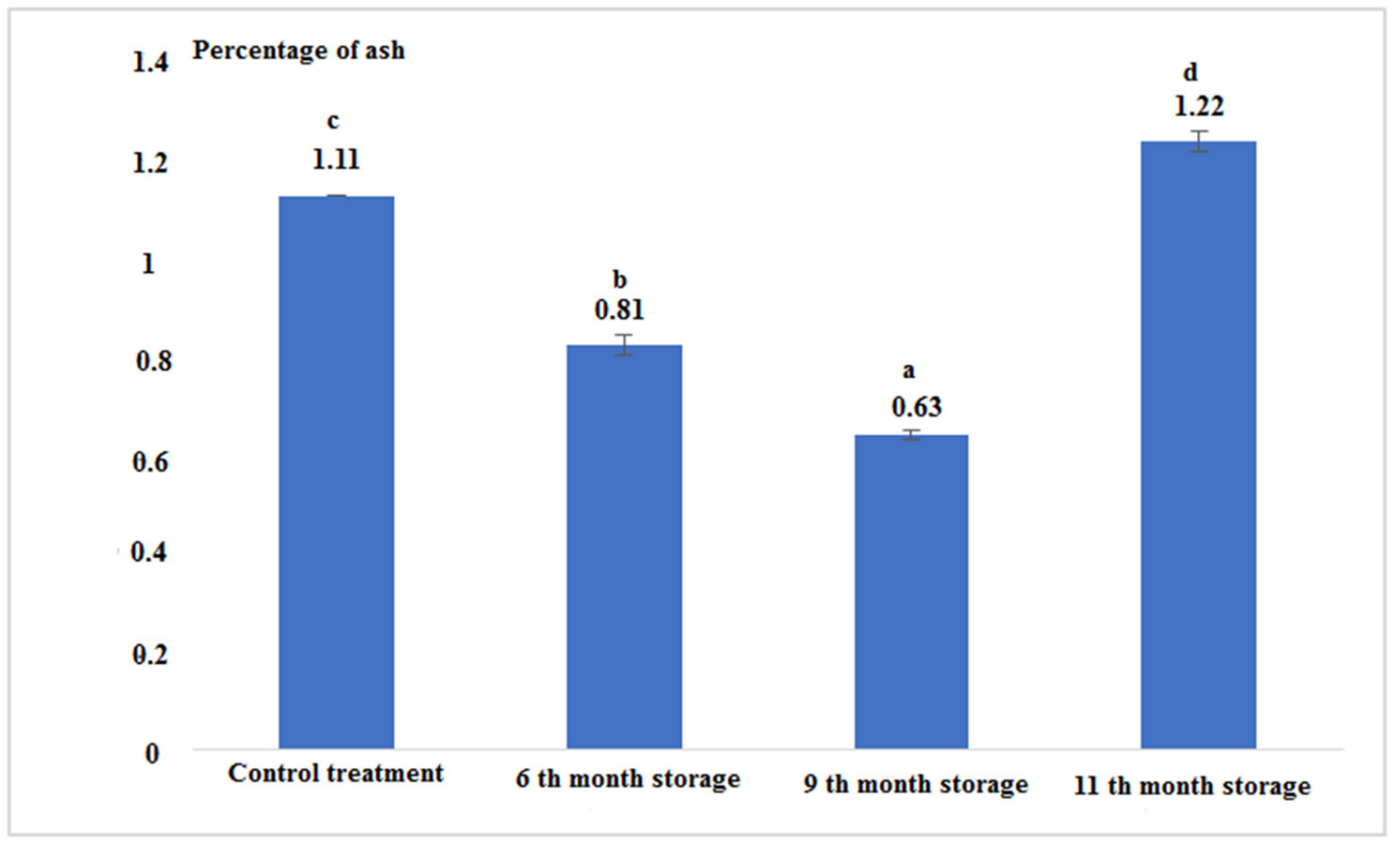
Percentage of the ash in fresh and canned tuna fillets.

The percentage of ash after the 6th and 9th month of storage was lower than the control sample. The highest percentage of ash was observed for the 11th month of storage. Statistical analysis of the data showed that there was a significant difference between all experimental treatments (*p* < .05; Figure 4 and Table 1).

**FIGURE 5:**
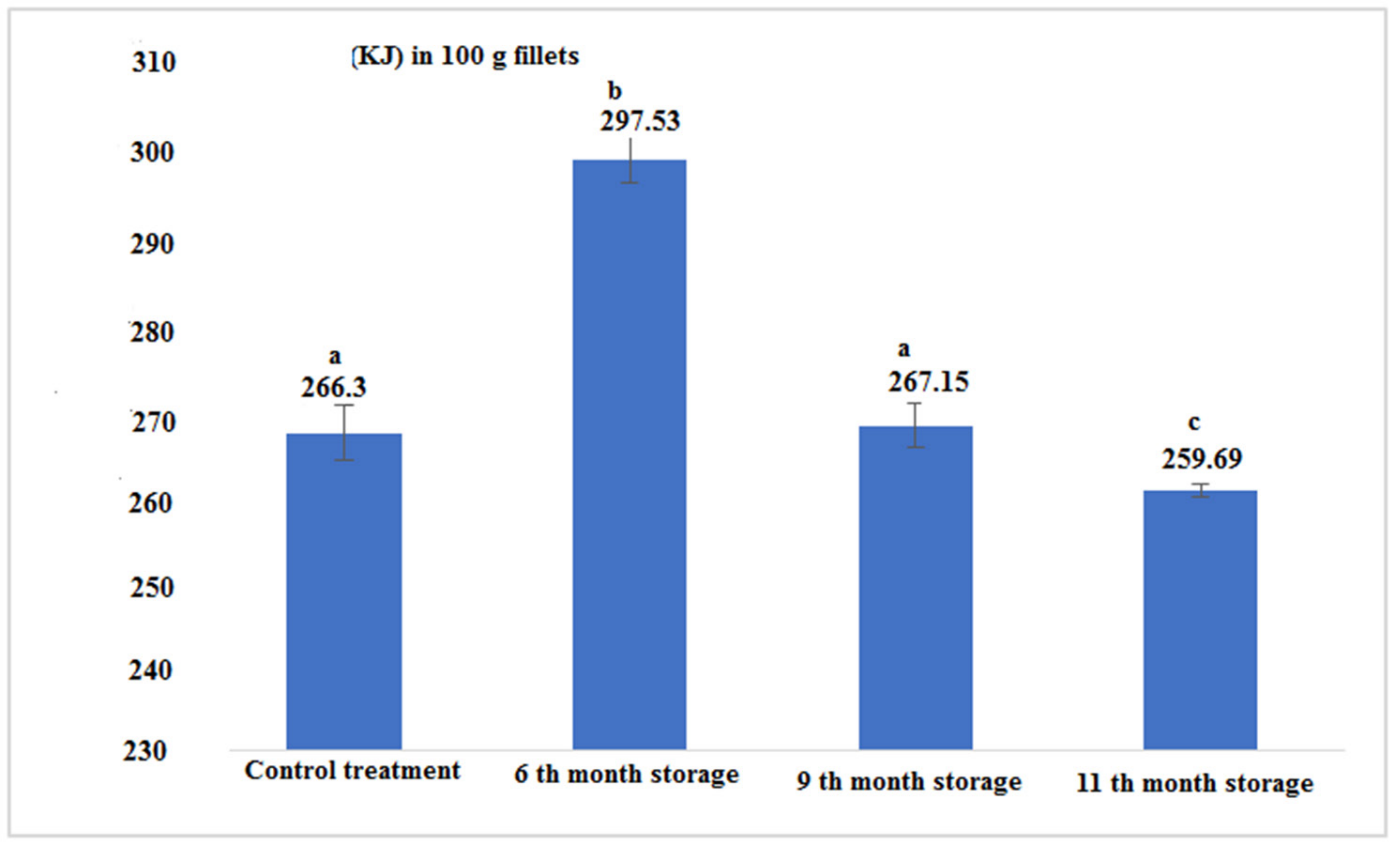
Energy value in the fresh and canned tuna fillets.

The energy value in canned tuna fillet after 6 months of storage was significantly higher than other experimental samples. The lowest energy level was found for the 11th month of storage. Statistical analysis of the data showed that there was no significant difference between the energy value in fresh fish fillets and canned fish in the 9th month of storage, but there was a significant difference between other treatments (Figure 5 and Table 1; *p* < .05).

**FIGURE 6:**
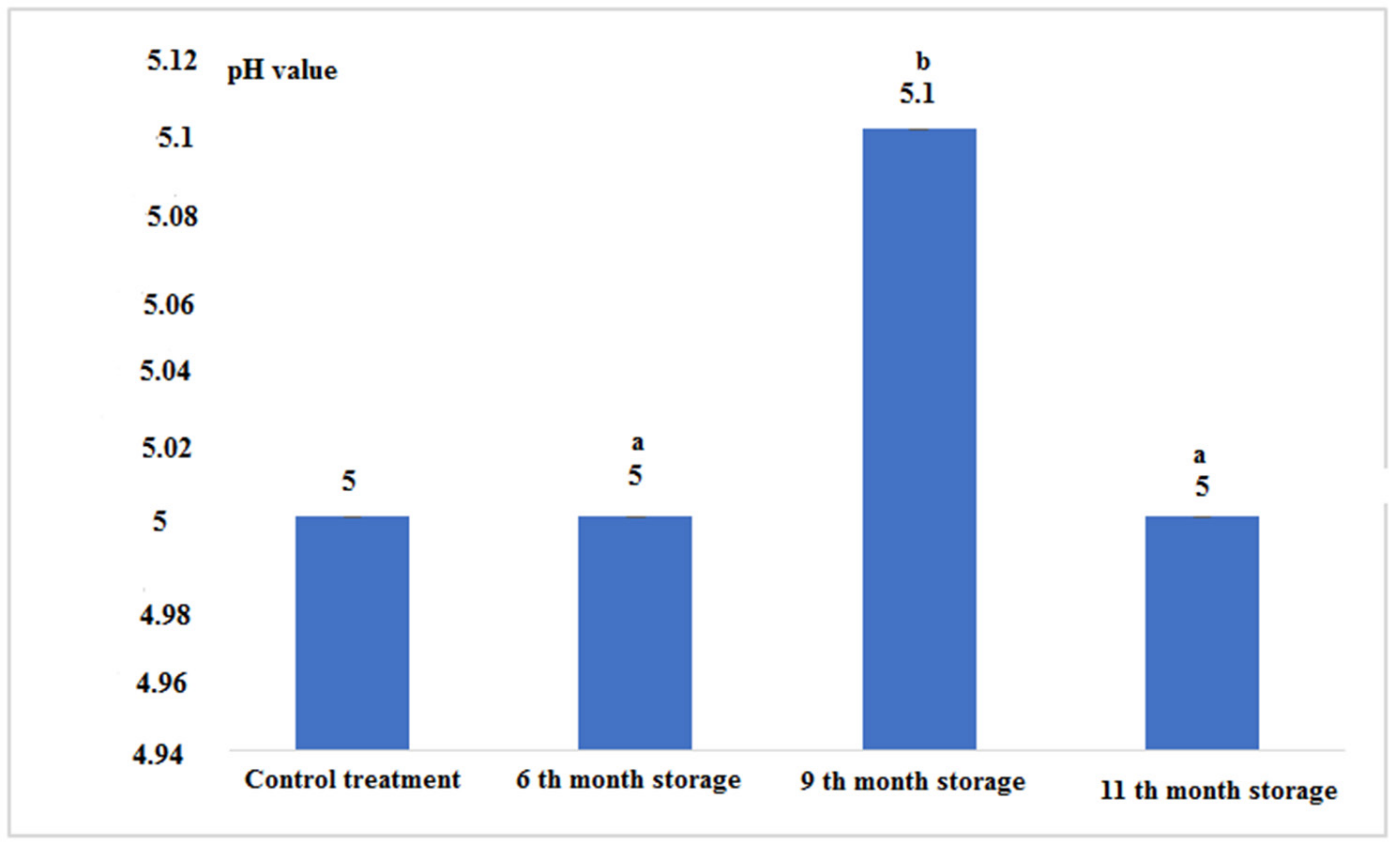
pH values in fresh and canned tuna fillets.

According to Figure 6 and Table 1, the pH level, except for canned fish in the 9th month of storage, which had the highest, in the other treatments had a constant value without significant difference (*p* < .05).

**FIGURE 7:**
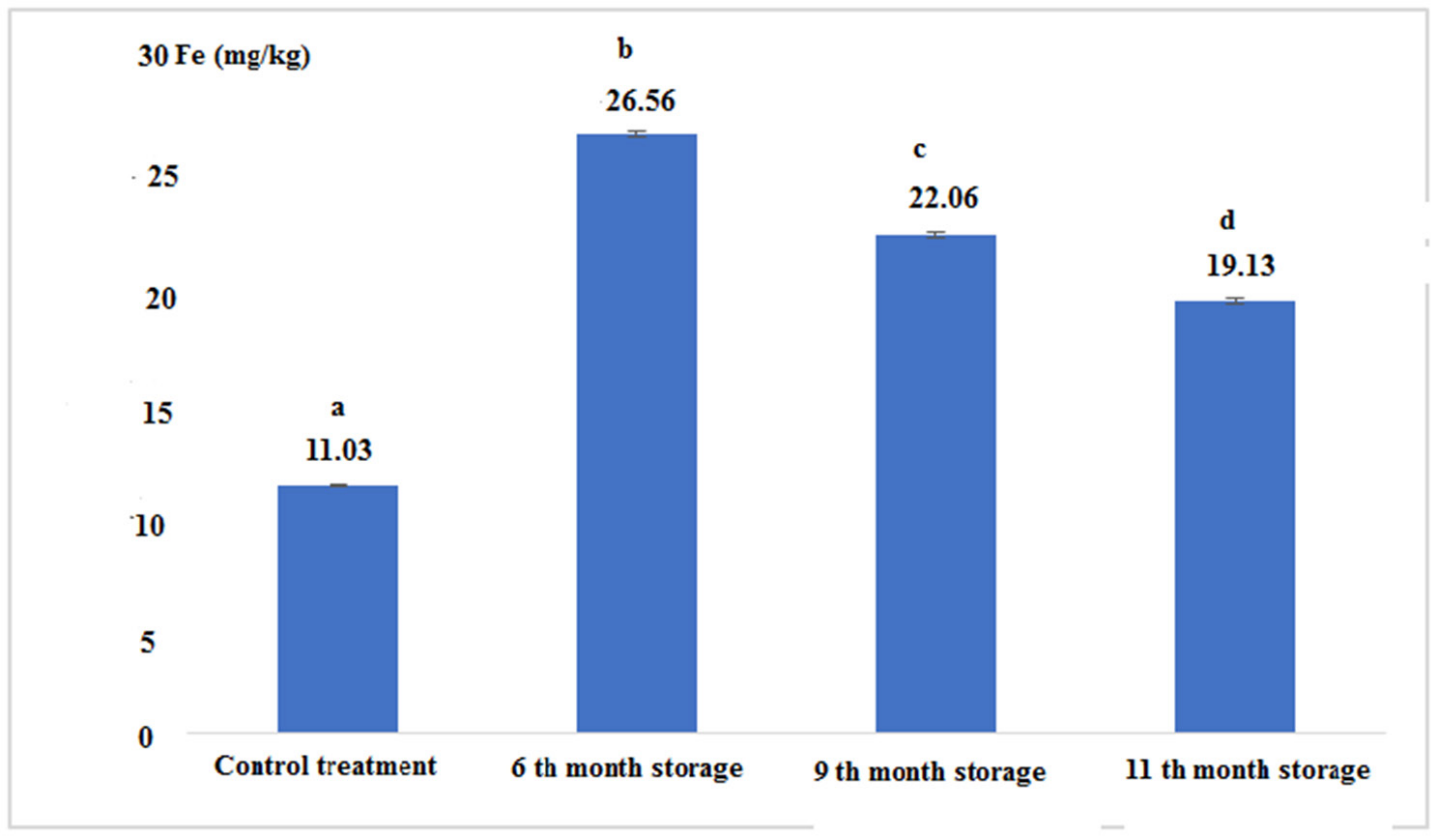
Concentration of the iron in fresh and canned tuna fillets.

**FIGURE 8:**
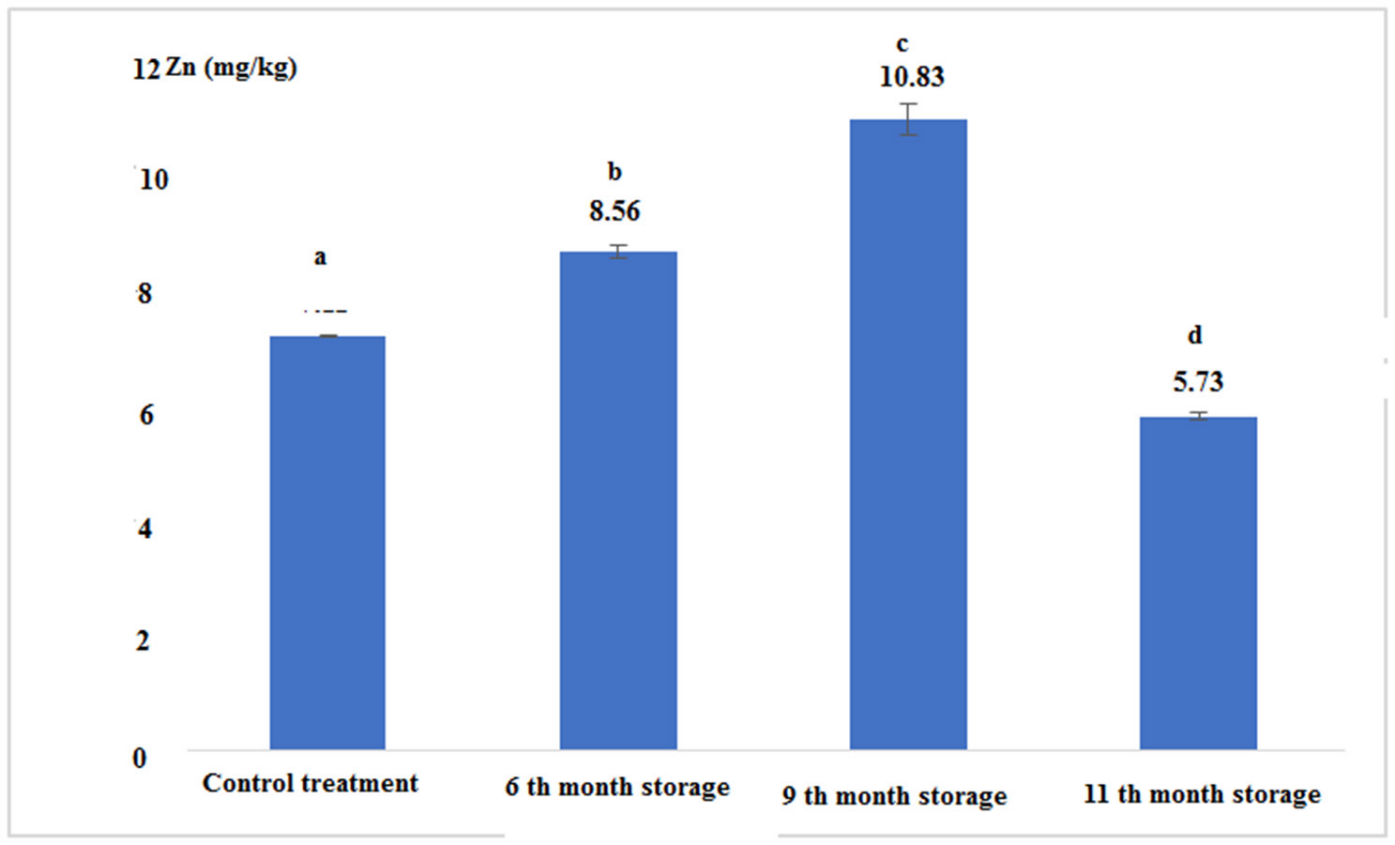
Concentration of the zinc in fresh and canned tuna fillets.

**FIGURE 9:**
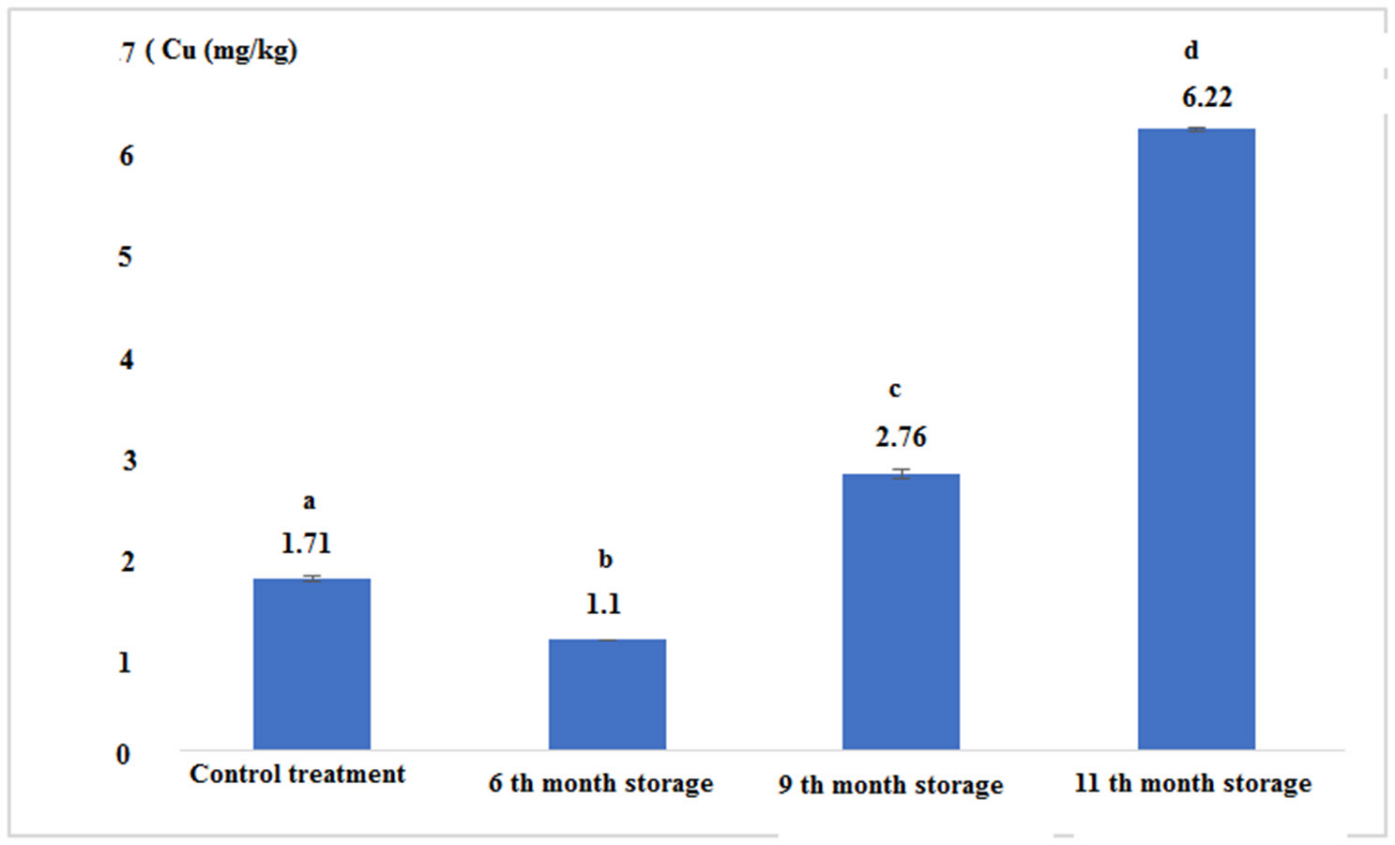
Concentration of the copper in fresh and canned tuna fillets.

**FIGURE 10:**
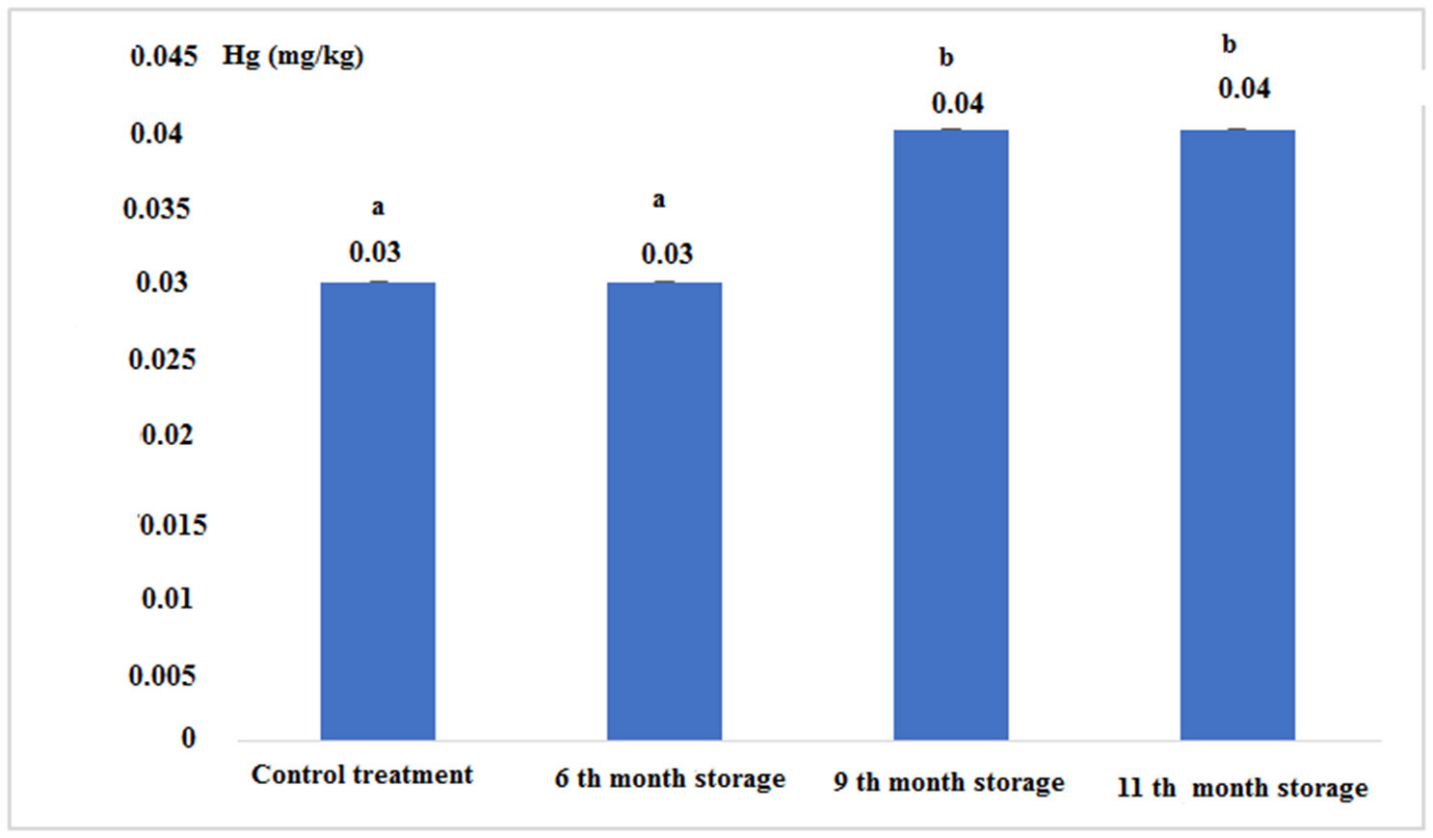
Concentration of mercury in fresh and canned tuna fillets.

The concentration of heavy metals in fresh and canned tuna fillets after 6, 9, and 11 months of storage is shown in Figures 7, 8, 9, 10:

Figure 7 shows that the iron concentration in canned fish in the 6th month of storage was higher than other experimental samples. The lowest content of iron was found for the control sample. There was a significant difference between all experimental treatments (*p* < .05).

Figure 8 and Table 2 show that there was a significant difference between the concentrations of zinc in all experimental treatments (*p* < .05). The highest was observed in the 9th month of storage with a concentration of 10.83 (mg/kg) and the lowest was observed in the 11th month of storage with a concentration of 5.73 (mg/kg).

**TABLE 2:** The concentration of heavy metals (mg/kg) in fresh and canned tuna fillets during different periods of storage.

Treatments	Fe	Zn	Cu	Hg
Control (Fresh)	11.03 ± 0.05^a^	7.11 ± 0.01^a^	1.71 ± 0.03^a^	0.03 ± 0.00^a^
6th‐month storage	22.56 ± 0.11^b^	8.56 ± 0.11^b^	1.10 ± 0.00^b^	0.03 ± 0.00^a^
9th‐month storage	22.06 ± 0.11^c^	10.83 ± 0.28^c^	2.76 ± 0.05^c^	0.04 ± 0.00^a^
11th‐month storage	19.13 ± 0.15^d^	5.73 ± 0.05^d^	6.22 ± 0.02^d^	0.04 ± 0.00^a^

*Note*: The same letters in each column indicate the absence of significant differences (*p* < .05).

Figure 9 and Table 2 show that the concentration of copper in canned tuna fillets over time significantly increased (*p* < .05). The concentration of this element in the control treatment was higher than the treatment 6th month of storage, but it was significantly lower than other experimental treatments. The highest copper content was observed in canned fish in the 11th month of storage (*p* < .05).

Figure 10 and Table 2 show that the concentration of mercury in the 9th and 11th month of storage increased compared to the 6th‐month storage, but this increase in the concentration was significantly different compared to control and 6th‐month storage (*p* < .05).

The results showed that decrease in moisture content in the control (52.5%) to 50.33% in the 9‐month storage led to an increase in fat content from 14.91% in the control to 18.1% and a decrease in ash content from 1.11% in the control to 0. 63% in treatment 9 months of storage, while the energy value in both treatments control and 9 months of storage without significant difference was approximately the same.

The concentrations of iron, copper, and mercury in the control treatment from 11.03, 7.11, 1.71, 0.03 mg/kg, respectively, increased to 22.06, 10.83, 2.76, and 0.04 mg/kg for 9‐month storage treatment. This indicated that the remained elements in the ash should be reduced after 9 months of storage.

With comparison of the moisture contents in treatments of control and 11 and 6 months of storage, it can be concluded that the moisture content has increased, the ash content has also increased, while the comparison of moisture in treatments of control and 9 months of storage showed that moisture content was low, and the ash content also was decreased. Among the studied elements, the iron and zinc content in the 6th‐month storage was increased, but the copper content was decreased and the mercury content did not change.

The reason for the increase in fat content in the treatment 6‐month storage was the exit of fat from the fish fillets, and the reason for the decrease in fat content in the treatments 9‐ and 11‐month storage was the fat breakdown. The reason for the decrease in the protein content in the treatments may be the degradation and denaturation of the proteins.

In the present study, the average concentration of mercury in the samples was 0.5 mg/kg which was lower than the global standard. Gochfeld and Burger (2004) evaluated 168 canned tuna in terms of mercury concentration. Their results showed that the average total mercury content in tuna fish was 0.456 mg/kg, while this total mercury content was 25% higher than the world standard. The maximum concentration of mercury obtained in their report was 0.956 mg/kg. Emami Khansari et al. (2002) stated that the mercury content in a sample of canned food in Iran was less than the standard. Although in the present study, the concentration of mercury in canned tuna increased over time, but its amount was lower than the values of international standards, and obtained results of the present study were in line with the results obtained from the study of other researchers.

In another study, the concentration of mercury of the analyzed canned fish was 146.65 ppb, which was lower than the global standards of EPA and FDA, while was stated that the permissible amount was 1 μg per g (Salar Amoli & Ali Isfahani, 2008) which was consistent with the results of the present study. Accumulation of heavy metals in the fish muscle varies according to ecological and biological conditions as well as the metabolic activities of the fish (Canli & Atli, 2002). A study by Ikem and Egiebor (2005) showed the concentration of copper metal in the analyzed canned fish was less than the MAFF standard, which was consistent with the results of the present study.

Velayatzadeh et al. (2010) investigated the heavy metals in some canned tuna in Iran. Their results showed that the highest concentration of iron was 7.63 ± 0.04 mg and the lowest concentration of iron was 2.84 ± 0.42 mg /kg. In the present study, the concentrations of zinc and iron in the canned fish showed a significant increase compared to the fresh sample. Zinc accumulates mainly in the bones and skin, but is also found in significant amounts in the liver, gills, and kidneys (Ismaili Sari, 2002). The place of absorption and the mechanism of its transfer to the fish body depends on factors such as the chemical form of the metal (ionic or its salts).

The canning process can change the concentration of heavy metals in the product. Atta et al. (1993) reported that the concentration of some heavy metals decreased during the cooking and frying process. A study by Ezzatpanah et al. (2009) showed that canning steps, including defrosting, baking, and sterilization, significantly reduced the concentration of heavy metals. In a recent study, the copper content in canned tuna in the 6th month of storage and the zinc content in the 11th month of storage was significantly reduced comparing to the fresh sample (*p* < .05).

Iron content had the highest among other elements measured in the fresh and canned tuna fillets, while mercury was the lowest concentration in the samples. These findings were consistent with other researchers' findings in which the iron content had the highest in different organs of the fish (Mahboob et al., 2016), which was in agreement with the present study results.

According to study results of Celik and Oehlenschläger (2007), the lowest and highest levels of zinc in canned fish in Turkey were in the range of 33.8–556 μg/g. Iron content in fish species obtained from Iskenderun, northeast of the Mediterranean Sea, Turkey, was reported in the range of 27.35–0.82 μg/g dry weight. The level of this element in Black Sea fish samples, Turkey, was in the range of 52.9–40.32 μg/g (Tuzen, 2003). One of the reasons for the different amounts of heavy metals in canned fish in different countries was the difference in the environmental conditions of ecosystems and the type of fish species (Table 3).

**TABLE 3:** Mean of element concentration (mg/kg wet weight) of present study canned tuna compared with other study results.

Fish species	Area	Tissue	Zn	Cu	Fe	Hg
Canned *Thunnus tonggol*	Present study	Muscle	8.37	3.36	22.58	0.03
Canned tuna	Iran	Muscle	7.31	2.30	6.66	0.17
Canned tuna	Nigeria	Muscle	4.63			
Canned tuna	Turkey	Muscle	33.8–556		27.35–0.82	
Canned tuna	Brazilin	Muscle	4.51 ± 0.16	0.94 ± 0.39	14.22 ± 6.4	
Canned tuna	Jordon	Muscle	26.20	2.21	13.5	
Canned tuna	Moroccan	Muscle				0.21
*Thunnus albacares*	Italy	Muscle				0.23 ± 0.02
Canned tuna	Libya	Muscle				0.29

Most species of tuna fish have a protein content in the range from 15% to 30%. Tuna fish contains low fat and calories, so it is a great alternative compared to meats and dairy products that contain saturated fats and trans fatty acids. The seafood proteins have valuable nutritional value. Fish protein contains all the essential amino acids and has highly digestible (Jhaveri et al., 1984). However, the amount of protein varies significantly between species which depends on size, sexual status, feeding season, and physical activity. Tuna contains a large amount of protein (27%) and is also rich in essential amino acids.

The fish body composition is a good indicator of its physiological condition, but it takes time relatively to measure. The proximate composition of the body includes the analysis of the contents of fish water, fat, protein, and ash. Carbohydrates and non‐protein compounds are available in small quantities and are generally regardless for analysis (Cui & Wootton, 1988). The percentage of water in the fish body is a good indicator of the relative contents of energy, proteins, and lipids. The lower the percentage of water, the higher the amount of lipids and proteins and the higher the energy of the fish (Dempson et al., 2004). According to the Food and Agricultural Organization (FAO; 2003), moisture and fat contents in fish fillets are inversely related, accounting for approximately 80%, with other components making up the remaining 20%. This inverse relationship has also been reported for marine fish species such as *Pseudosciaena aeneas* and *Johnius carutta*, *Mullus barbatus* (Lioret et al., 2007; Rao & Rao, 2002).

In the present study, this relationship was also observed. Aberoumand (2014) study showed that the percentage of protein in fresh yolk tuna fillet was lower than canned fish after 2 months of storage, but with over time, the percentage of protein in canned fish fillet decreased compared to the control. The fat percentage in canned fish was higher than in fresh samples. The percentage of canned fish ash in the 2nd and 6th month of the experiment was lower than the control, but in the 4th month of storage was significantly increased. The pH of canned samples was also higher than the pH of fresh fish fillets. The energy value of canned fish increased significantly with over time. Aberoumand (2011) study on the Havoor tuna fish reported that the percentage of fat, energy, and pH of canned Havoor fish increased significantly compared to the fresh Havoor, but the percentage of ash and protein decreased during the canning process. In canned Zardeh tuna fish, the percentage of moisture decreased during the experiment. In agreement with these results, in the present study, the percentage of fat in canned fish increased significantly compared to fresh samples and the percentage of protein decreased significantly. Changes in moisture and ash contents fluctuated. The energy value in the fillets in canned fish was higher than in the fresh sample, but with over time its value decreased.

Otto Santa (1996), in another study, entitled changes in the nutritional quality of Albaco fish after canning, stated that the percentage of moisture and protein decreased during the canning process, but the percentage of fat increased. In the present study, the percentage of fat increased from 14.91% in the fresh sample to 21.03% in the 6th‐month storage and then decreased to about 17.53% in the 11th month of the experiment, but in all samples, this percentage was higher than fresh fish fillets.

Souci et al. (2000) reported that moisture, protein, fat, and ash contents of canned fish fillets after the sterilization process were 52.5%, 23.8%, 20.9%, and 2.3%, respectively, which were consistent with the results in the present study. The reaction of a mixture of water and oil with nutrients, especially at high temperatures during the canning process, led to change the structure of the oil and the nature of nutrients, such as proteins (Kubow, 1992), which led to the significant difference in the moisture content in different samples. In addition, the place and season of fishing, fish size, sexual maturity, and spawning period, affect the amount of fat, moisture, and protein of the fish (Kubow, 1992).

The lowest percentage of moisture in the present study was observed for the 9th‐month storage, which was significantly lower than the fresh sample (*p* < .05), while Allen (1987) and Frankel (1991) considered the reduction of moisture content during processing as an advantage because it reduced fish sensitive to microbial spoilage and oxidative degradation of unsaturated fatty acids which thus improved the nutritional value of the fish at long time during storage.

The fish species contain high‐fat content with a high nutritional value, because of their omega‐3 fatty acids, which support a protective effect against coronary heart disease (Alonso et al., 2003). In the present study, the fat content in the canned fish was higher than fresh fish. One of the reasons for the high percentage of fat in canned samples was the use of oil as filler in canned food. Garcıa‐Arias et al. (2003) also reported that in cooked fish fillets, the moisture content decreased and the fat content increased. Ash content indicates the amount of minerals in each food, such as fish (Omotosho et al., 2011). Concentrations of minerals and trace elements in total ash in fish vary depending on feeding behavior, increase weight or length, fishing season, environment and ecosystem, and migration even in the same area (Abdallah, 2007; Canli & Atli, 2002). Ash content changes during storage due to moisture absorption and protein loss (Hassan et al., 2013). The smaller fish species show higher ash content due to higher bone to meat ratio (Daramola et al., 2007), so the large size of tuna fish can be considered as a reason for the low percentage of ash in the present study.

In the present study, pH levels in all samples except for the 9th‐month storage were not significantly different from each other (*p* < .05). The pH of canned tuna increased significantly after 9 months of storage (*p* < .05). The higher pH values in canned samples may be due to the formation and accumulation of some amino acids and volatile nitrogen compounds such as NH_3_ as a result of the breakdown and proteolysis of proteins during heat treatment (El‐Sherif, 2001). Czerner et al. (2015) studied the effect of canning process on the physicochemical properties of *Engraulis anchoita* and found that the pH of the fresh fish was 6.07 which after canning and during storage at room temperature due to the breakdown of protein into basic products such as ammonia, amines, and hydrogen sulfide which increased to 6.12.

El‐Lahamy and Mohamed (2020) reported that canning process decreased the protein content for *Orcynopsis unicolor*, but increased the protein content for *Euthynnus affinis*. El‐Dengawy et al. (2012) reported the chemical composition of 16 samples of canned fish (canned tuna, canned sardine, canned Mackerel) and observed that moisture percentage in all canned fish samples ranged between 52.41 ± 0.035% to 78.53 ± 0.142%, which was in agreement with the present study. Sajib et al. (2015) studied the effect of canning process on the chemical composition of chela (*Laubuka dadiburjori*) and reported that moisture, protein, lipid, ash, and carbohydrate contents of fresh fish were 76.56 ± 1.62%, 13.74 ± 1.22%, 4.25 ± 0.85%, 2.37 ± 0.56%, and 1.41 ± 0.79%, respectively. After canning process, these contents changed to 67.15 ± 1.69%, 16.68 ± 0.88%, 5.46 ± 0.34%, 8.15 ± 0.83%, and 1.35 ± 0.07% for moisture, protein, lipid, ash, and carbohydrate, respectively, while in the present study, moisture after 9 months of storage changed from 52.5% in fresh sample to 50.33%. The ash content after 11 months of storage changed from 1.11% in fresh sample to 1.22%. The protein content changed from 22.66% in fresh sample to 18.33% in the final product after 11 months of storage. The fat content after 6 months of storage changed from 14.91% to 21.03% (Aberoumand & Baesi, 2022).

The high content of metals in Iranian canned fish in the present study may be due to environmental pollution and raw materials or due to secondary pollution such as improper handling of raw materials, containers, and processing steps on land and/or at sea. Because the percentage of heavy metals in the examined canned fish and fresh tuna was lower than the limit recommended by national and international organizations, there is no human risk assessment index for the heavy metals.

Chemical pollution (heavy metals) enters the body of some fish species from the polluted marine environment and accumulates in the fish meat; therefore, this type of fish that was processed and canned has an adverse effect on the nutrition and health of the consumers. Because the concentration of any of the heavy and toxic metals in canned fish was lower than the permissible international and Iranian limits, it should not report to the Iranian health authorities to prevent the distribution of these products in the markets and the canned fish limited consumption will not be harmful for human consumption.

## 4 CONCLUSION

The increase in fat content in the 6th month of storage was due to the addition of oil to the canned fish and its absorption, but the decrease in fat content in the 9th and 11th months of storage was probably due to the oil breakdown. The decrease in protein content in the canned fish was probably due to protein denaturation in thermal processing. Analysis of the chemical composition of the samples also showed that although the protein content of canned samples was reduced compared to the fresh fish, overall, the nutritional value of canned fish was maintained at an acceptable level, which provides the human body needs for these nutrients. Because of water content and analytical issues changed, the concentrations of heavy and toxic elements changed in canned tuna fish. The concentrations of the heavy metals iron, zinc, copper, and mercury in canned tuna fish were higher than in fresh tuna fillets, but due to that, these amounts were less than the permitted levels and international standards, its limited consumption will not be harmful for human consumption. Therefore, the consumption of canned Iranian tuna can be safe for human health despite the possible contamination with heavy metals.

## FUNDING INFORMATION

This study was funded by the Behbahan Khatam Alanbia University of Technology, Behbahan, Iran.

## CONFLICT OF INTEREST STATEMENT

The authors do not have any conflict of interest.

## Data Availability

The datasets used and/or analyzed during the current study are available from the corresponding authors on reasonable request.
